# COVID-19 transmission risk and protective protocols in dentistry: a systematic review

**DOI:** 10.1186/s12903-020-01270-9

**Published:** 2020-10-08

**Authors:** Morteza Banakar, Kamran Bagheri Lankarani, Dana Jafarpour, Sedigheh Moayedi, Mohammad Hasan Banakar, Ashkan MohammadSadeghi

**Affiliations:** 1 Health Policy Research Center, Institute of Health, Shiraz University of Medical Sciences, Shiraz, Iran; 2 Biomaterials Research Center, School of Dentistry, Shiraz University of Medical Sciences, Shiraz, Iran; 3 Department of Orthodontics, Mashhad University of Medical Sciences, School of Dentistry, Mashhad, Iran; 4 School of Dentistry, Yasuj University of Medical Sciences, Yasuj, Iran; 5 School of Pharmacy, Shiraz University of Medical Sciences, Shiraz, Iran

**Keywords:** COVID-19, SARS-CoV-2, Dentistry, Dental practice management, Dental public health, Infection control

## Abstract

**Background:**

Among several potential transmission sources in the spreading of the COVID-19, dental services have received a high volume of attention. Several reports, papers, guidelines, and suggestions have been released on how this infection could be transmitted through dental services and what should be done. This study aimed to review the guidelines in order to develop a practical feasibility protocol for the re-opening of dental clinics and the reorientation of dental services.

**Methods:**

This study systematically reviewed the published literature and the guidelines of international health care institutions on dentistry and COVID-19. We searched Pubmed, Web of Science, and SCOPUS electronic databases using MESH terms. The recommendations identified were tested with a convenience sample of experienced practitioners, and a practical step-by-step protocol is presented in this paper.

**Results:**

To the date this paper was drafted, 38 articles were found, of which 9 satisfied our inclusion criteria. As all the nine studies were proposed in a general consensus, any elective non-emergency dental care for patients with suspected or known COVID-19 should be postponed for at least 2 weeks during the COVID-19 pandemic. Only urgent treatment of dental diseases can be performed during the COVID-19 outbreak taking into consideration pharmacological management as the first line and contagion-reduced minimally invasive emergency treatment as the secondary and final management.

**Conclusions:**

While the currently available evidence has not demonstrated a clear and direct relationship between dental treatment or surgery and the possibility of the transmission of COVID-19, there is clearly the potential for transmission. Therefore, following the protective protocols in the COVID-19 crisis is of utmost importance in a dental setting.

## Background

COVID-19, the newly discovered coronavirus disease first diagnosed in China in 2019, has become pandemic throughout the world in a relatively short period of time. It has affected almost all aspects of human life worldwide. Many protocols have been established to minimize the number of infected people, yet this virus has already spread to all five continents, affecting all communities regardless of borders, nationalities, or climate conditions [1, 2]. Up to May 31, 2020, the number of people who were officially reported to have been infected by COVID-19 around the globe was more than 5,934,936 victims, out of which 367,166 deaths have been reported [3]. It seems that the actual numbers might be much higher than the above figures.

Fever, cough, and fatigue have been reported as the major clinical symptoms of the infection following a median incubation period of 3 days, with a great number of infected patients also showing impairment in smell (anosmia) and taste (ageusia) as vital clinical findings in early diagnosis of COVID-19 infection [4]. COVID-19 has similar transmission pathways, but not identical, to those of other SARS-CoV infections, being mainly through the respiratory system [5, 6]. Many considerations regarding possible hazardous activities or workplaces have been raised based on both our experience from previous SARS-CoV infections and our observation of the transmission pattern of the SARS-CoV-2 itself in such a short time following its appearance. Among them, the potential transmission of the virus through dental procedures and in dental settings has attracted much attention leading to either mandatory or voluntary suspension of routine dental care [7, 8].

The concern about dental practice coronavirus transmission has been widely recognized around the world. Recently, The New York Times noted that dentistry was the most at-risk profession for nCoV-19 compared to other various occupations [9]. Based on the nature of the dental procedures, and the proximity of the dental team with patients, the disease could readily spread from infected patients to the dental team, and vice versa, and subsequently to other patients, if appropriate protective infection control measurements are not undertaken [10, 11].

Dental teams, led by the dentist, are very familiar with universal personal protective equipment and other cross infection control measures and risk assessment. While these issues have become prominent during the pandemic, there has been uncertainty regarding the most appropriate Personal Protective Equipment (PPE) and way of working. Each country of the world has been required to develop policies to counter COVID-19 rapidly and has interpreted medical and scientific evidence and advice from the WHO in very different ways. Similarly, the guidelines written for COVID-19 and advice published for the safe and effective practice of dentistry have shown much variation around the world and also within countries. Perhaps this is due to the lack of evidence-based research on the efficiency of the proposed guidelines. It is well known that developing an effective vaccine that implements widespread immunization takes time; therefore, it is critical to find new practical methods in our daily dental practice so that we can offer much-needed care for patients with oral health issues. The long term consequences of this pandemic are currently unknown, but they will undoubtedly result in a ‘new normal’ for the provision of dental care.

Many suggestions and protocols have been issued for the re-opening of or reorientation of dental clinics over a short period. However, many of the protocols have been produced posthaste (for understandable reasons) with a focus on the ideal rather than a realistic point of view [12–14]. This systematic review focuses on the risk of the transmission of the COVID-19 during dental treatment and provides pathways and protective protocols to minimize it, bearing in mind the long-term necessity of actions and realistic, practical measures.

## Method

**Fig. 1:**
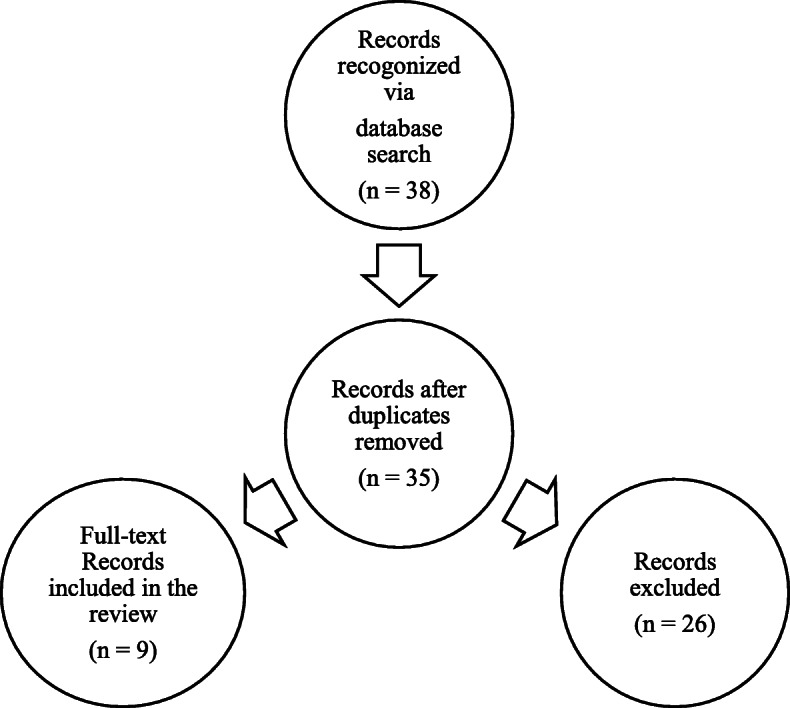
Systematic review flow chart

In this systematic review of the literature, we searched Pubmed, ISI, and SCOPUS electronic databases using MESH terms and the following keywords: (“Covid-19” OR “Covid19” OR “Corona” OR “Coronavirus” OR “SARS-CoV-2”) AND (“Dentistry” OR “Dental”). All articles from the 01.01.2020 until 10.05.2020 that satisfied our selection criteria of being recommendations or guidelines for dental practice during the COVID-19 pandemic were retrieved. Articles were excluded if they were not found to be relevant, produced before the COVID-19 pandemic, or opinion-based without any supporting evidence. Some clinical organizations for example; The World Health Organization (WHO), The Centers for Disease Control and Prevention (CDC), The National Health Service (NHS), The American Dental Association (ADA) and, American Dental Hygienists’ Association (AHDA) had also published recommendations and guidelines through their websites. Therefore, we also undertook a Google search for these sources and used the English, German, and Farsi languages. We outlined the competency criteria using a PICO model as follows: Population: All articles and/or guidelines for dental practice during the COVID-19 pandemic. Intervention: Knowing and applying the special precautions and therapeutic considerations in dentistry. Comparison: Despite the fact that due to the novelty of COVID-19, it is difficult to compare the effectiveness and applicability of the proposed precautions and interventions, but the comparisons have been made based on country, and organization of the article and guidelines. Outcome: the application and effectiveness of the intervention for reducing risks of transmission. Figure 1 shows a flow chart of the literature screening method used. These were reviewed by two authors independently, who have experience in infection control in dentistry and medical study methodology. Articles were critically appraised and data extracted to compile a summary clinical protocol for dental practice during the COVID-19 pandemic. The extracted statements of recommendation, both from the published articles and the clinical organization’s publications, were divided into grouped items. We also added to this grouping by including the views of ten dentists with more than 5 years of clinical work experience in addition to the views of the two authors who had undertaken the data extraction. These additional dentists were selected conveniently based on clinical experience from Iran.

## Main results

To the date this paper was drafted, 38 articles were found, of which 9 satisfied our inclusion criteria. The key feature of the nine included studies are summarized in (Table 1). We noted that some researchers preferred to publish their work rapidly and in alternative ways of using peer-reviewed journals.

**Table 1:** An overview of the guidelines provided for Coronavirus 2019 in dentistry by articles

I.D.	Author (Year)/ Country	Guidelines offered	Procedural considerations	Authors’ comments on the literature gap
1	**Alharbi et al. (2020)/ Saudi Arabia** [15]	I.) Emergency Tx (fracture and infection compromising patient’s airway, uncontrolled bleeding) for all patients	I.) Restrict Intraoral imaging	-Lack of a guideline for patients who need dental Tx before an imminent transplant.
			II.) Preprocedural use of 0.23% povidone-iodine mouth-wash at least 15 s	
		II.) Minimally invasive urgent care without aerosol generation for asymptomatic suspect, stable active and recovered patients		-Lack of a guide on proper saliva ejectors or surgical aspiration
			III.) Single-use devices	
			IV.) Use a rubber dam	
		III.) Invasive urgent care with aerosol generation for asymptomatic suspect	V.) Avoid aerosol-generating procedures	
			VI.) Avoid administering Ibuprofen	
2	**Ather et al. (2020)/ The United States** [14]	I.) Perform dental Tx if lack of travel hx/epidemiological link	I.) Personal protective equipment and hand hygiene	-Lack of a guideline for patients who need dental Tx before an imminent transplant.
		II.) Defer elective care for suspect at least 2 weeks		
				-Lack of a guide on proper saliva ejectors or surgical aspiration
		III.) Urgent care for suspect in case of tooth pain and/or swelling using pharmacological management as the first line and emergency care as the secondary management	II.) Preprocedural mouth rinse	
			III.) single-use devices	
			IV.) Avoid Intraoral radiography	
			V.) Use a rubber dam	
			VI.) Minimize ultrasonic instruments, high-speed handpieces, and 3-way syringes	
			VII.) Dilute Naocl to 1%	
			VIII.) Negative-pressure treatment rooms	
			IX.) Disinfect inanimate surfaces	
3	**Izzetti et al. (2020)/ Italy** [16]	I.) Identify potentially at-risk cases and support them in contacting the health authorities	I.) 1-min mouth rinse with 0.2 to 1% povidone, 0.05 to 0.1% cetylpyridinium chloride, or 1% hydrogen peroxide	-Lack of a precise guideline on the management of patients at various stages of the disease, from positive to asymptomatic to healed ones.
		II.) Understand the real need for professional consultation and preferably address the issue with just pharmacologic prescription		
			II.) Hand washing for at least 60 s and then hand rubbing with 60-70% hydroalcoholic solution before wearing a glove	
		III.) Organize a contagion-reduced treatment for the subjects with unknown risk of contagion who are experiencing an acute dental problem that requires immediate treatment		
			III.) Personal protective equipment	
			IV.) Preparation of all instruments in advance	
			V.) Total protection through disposable cover	
			VI.) Avoid, when possible, use of handpieces/ultrasonic instruments	
			VII.) Use a rubber dam	
			VIII.) Surgical aspiration system	
			IX.) If possible, prefer 4-hands technique	
			X.) Limit overall Tx time if possible	
4	**Lee and Auh (2020)/ Korea** [17]	I.) Routine pre-check the general health status and travel history to epidemic areas	I.) Use basic personal protective equipment for potential asymptomatic carriers	-Lack of a precise guideline on the management of patients at various stages of the disease, from positive to asymptomatic to healed ones.
		II.) Patients with suspected or known COVID-19 should be isolated or postpone their non-emergency dental care during the COVID-19 pandemic	II.) Hand washing is essential	
			III.) Must avoid or minimize procedures producing droplets or aerosols or stimulate salivary secretion or coughing.	
			IV.) Use high-volume saliva ejectors with the four-handed technique	
			V.) Minimize using the three-way syringe	
			VI.) Acquisition of extraoral radiographs rather than intraoral radiographs	
			VII.) Use an oxidative or antimicrobial mouth rinse before dental procedures	
			VIII.) Treatment in an isolated and well-ventilated environment	
			IX.) Disinfect the surface of equipment with 62–71% ethanol before and after dental procedures	
5	**Mallineni et al. (2020)/ Saudi Arabia- The United Kingdom- The United States-Brazil** [18]	I.) Contemporary minimally invasive procedures that minimize or eliminate aerosol generation should be employed where intervention is indicated throughout the pandemic	I.) Hand hygiene	-Lack of a precise guideline on the management of pediatric patients at various stages of the disease, from positive to asymptomatic to healed ones.
			II.) Personal protective equipment	
			III.) Respiratory hygiene/cough etiquette	
		II.) Once restrictions begin to be eased, continue management of dental disease with minimally interventive concepts, e.g., atraumatic restorative treatment, fissure sealants, silver diamine fluoride, selective caries removal, and the Hall Technique while viral transmission risk remains high	IV.) Sharps safety and safe injection practices	
			V.) Sterilization and disinfection of patient-care items and devices	
			VI.) Environmental infection prevention and control	
			VII.) Dental unit water quality	
6	**Meng and Hua (2020)/ China** [19]	I.) In areas where COVID-19 spreads, non-emergency dental practices should be postponed.	I.) Hand hygiene	-Lack of a precise guideline on the management of patients at various stages of the disease, from positive to asymptomatic to healed ones.
			II.) Personal protective equipment	
		II.) Pulp exposure in symptomatic irreversible pulpitis could be made with chemomechanical caries removal.	III.) Thorough disinfection of all surfaces	
			IV.) Particulate respirators (e.g., N-95 masks or FFP2)	
		III.) If a tooth needs to be extracted, an absorbable suture is preferred.		
		IV.) For patients with facial soft tissue contusion, debridement, and suturing should be performed.	V.) The 4-handed technique is beneficial	
			VI.) Use saliva ejectors with low or high volume	
		V.) Life-threatening cases with oral and maxillofacial compound injuries should be admitted to the hospital immediately.		
			VII.) Preoperative antimicrobial mouth rinse	
			VIII.) Minimize aerosol-generating procedures, such as the use of a 3-way syringe	
			IX.) Acquisition of extraoral radiographs rather than intraoral radiographs	
			X.) Rubber dam	
			XI.) Isolated and well-ventilated room or negatively pressured rooms if possible	
7	**Peng et al. (2020)/ China** [20]	I.) If a patient replies “yes” to screening questions, and body temperature is below 37.3 °C, the dentist can defer the treatment until 14 days after the exposure event.	I.) Hand Hygiene	Lack of a precise guideline as to which dental treatments can be performed in case the patient replies “no” to all screening questions and his/her body temperature is below 37.3 °C
			II.) Personal protective measures for the dentists	
		II.) If a patient replies “yes” to screening questions, and body temperature is no less than 37.3 °C, the patient should be immediately quarantined and reported to the infection control department.	III.) A Preprocedural mouth rinse containing oxidative agents such as 1% hydrogen peroxide or 0.2% povidone especially when a rubber dam cannot be used	
		III.) If a patient replies “no” to all screening questions, and his/her body temperature is below 37.3 °C, the dentist can treat the patient with extra- protection measures and avoids spatter or aerosol-generating procedures.		
			IV.) If using a rubber dam, use extra high-volume suction for aerosol and spatter along with regular suction with a four-hand operation	
		IV.) If a patient replies “no” to all screening questions, but his/her body temperature is no less than 37.3 °C, the patient should be instructed to specialized clinics for COVID-19.		
			V.) If a rubber dam isolation is not possible, manual devices, such as Carisolv and hand scaler, are recommended for caries removal and periodontal scaling	
			VI.) the use of dental handpieces without anti-retraction function should be prohibited during the epidemic period of COVID-19	
			VII.) Disinfection of the clinic settings	
8	**Prati et al. (2020)/ Italy** [21]	I.) Triaging patients to detect by history and with a respiratory infection, flu, acute respiratory illness, conjunctivitis, and cardiovascular abnormalities	I.) Regular, meticulous and effective hand wash	The study provides a guideline for dental school; however, more precise guides on the management of patients at various stages of the disease, from positive to asymptomatic to healed ones, are required.
		II.) Separation of patients with respiratory symptoms to limit their contact with the dental staff, students and patients	II.) Use face masks	
			III.) Decontamination of all surfaces with 0.1% sodium hypochlorite or 70% ethanol or 0.5% hydrogen peroxide	
		III.) Avoiding dental treatment if at all possible		
			IV.) Respiratory hygiene/cough etiquette	
			V.) Isolate the patient in a dedicated single-patient room (with closed door)	
			VI.) Use a rubber dam	
			VII.) Application of powerful air/water surgical suction pump (aspirator) close to the tooth and a second suction close to the nose to prevent aerosol and saliva droplet diffusion	
			VIII.) Use high-speed handpiece with no exhaust	
			IX.) Decontamination of equipment, surgeries/ operatories after each patient	
9	**Spagnuolo et al. (2020)/ Italy** [22]	I.) Dentists should avoid the scheduling of any patient: only such urgent dental diseases can be considered during the COVID-19 outbreak.	I.) Staff should work at an adequate distance from patients	-Lack of a precise guideline as to which dental Tx should be considered as urgent dental disease
			II.) Handpieces must be equipped with anti-reflux devices to avoid contaminations	
			III.) Avoid or minimize operations that can produce droplets or aerosols	
			IV.) Use of saliva ejectors with a low volume or high volume	

As all the nine studies were proposed in a general consensus, any elective non-emergency dental care for patients with suspected or known COVID-19 should be postponed for at least 2 weeks during the COVID-19 pandemic. Only urgent treatment of dental diseases can be performed during the COVID-19 outbreak taking into consideration pharmacological management as the first line and contagion-reduced minimally invasive emergency treatment as the secondary and final management.

Furthermore, some of the guidelines provided by specific clinical organizations were reviewed. Since these protocols were very long, they were summarized, and the key elements were extracted from these published guidelines. The recommendations and guidelines identified are shown in Table 2.

**Table 2:** Guidelines that should be adopted in a dental setting during COVID-19 [3, 23–29]

Prior to dental treatment	Before entering a dental office	- Delay non-urgent dental and cosmetic services.	ADA, CDC, ADHA, NHS
		-Prevent crowding in appointment setting by booking appointments.	ADA
		-Dental procedures in patients with a history of COVID-19 should be postponed for at least 1 month.	WHO
		-High-risk patients like diabetic and immunocompromised patients are treated at the early hours of a dental office opening.	NHS
		-Use telephone triage, teleconferencing, or Teledentistry options as alternatives to in-office care, if possible.	CDC, NHS, ADA
		- Ask staff to stay home if they are sick.	CDC, ADA
		-Actively screen and record the temperature of each staff. Send staff home if they develop symptoms while at work.	CDC, NHS
	At dental office	-Actively screen the patient at the time of check-in. Patients with fever should refer to specific medical centers. If the patient is afebrile (temperature < 100.4 °F) and otherwise without symptoms consistent with COVID-19, then emergency dental care may be provided.	CDC
		-No accompanying individuals should be allowed.	CDC, ADA
		-Offer hand wash or hydroalcoholic solutions (with 60–75% alcohol) for hand disinfection upon entrance to the dental office.	NHS, ADA
		-Provide a large room with adequate ventilation in the waiting area.	NHS
		-Appropriate zoning and separation measures should be undertaken. Waiting rooms and reception areas should allow for 2-m separation, ideally marked on chairs and flooring.	NHS
		-Remove magazines, reading materials, toys, and other objects that may be touched by others and which are not easily disinfected.	ADA
		- Place signage in the dental office for instructing patients on standard recommendations for respiratory hygiene/cough etiquette and social distancing.	ADA
		- Require the use of facemasks or cloth face coverings by everyone entering the dental office	CDC
		- Dental professionals should implement PPE (isolated wearing like N-95 masks, Health or FFP2-standard masks, gloves, face shields, goggles, gown, surgical cap, shoe cover)	CDC, NHS, ADA
		-Preparation of materials and instruments in advance and cover surfaces with disposable protections	NHS
		-Materials stored in a refrigerator should be sterilized before and after each treatment	WHO
		-Patients should be treated in an isolated and well-ventilated room with negative pressure relative to the surrounding area	CDC
During dental treatment		-Hand hygiene should be performed before and after all patient contact, contact with potentially infectious material, and before putting on and after removing PPE.	CDC
		-Use alcohol-based hand rub (ABHR) with 60–75% alcohol. If hands are visibly soiled, use soap and water for at least 20 s before returning to ABHR.	
		-Preoperative antimicrobial mouth rinse like peroxide could reduce the number of microbes in the oral cavity. Since SARS-CoV-2 may be vulnerable to oxidation, use 1.5% hydrogen peroxide or 0.2% povidone as a preprocedural mouth rinse.	ADA
		-Rubber dams and high-volume saliva ejectors can help minimize aerosol or spatter in dental procedures.	CDC, NHS, ADA
		-use extraoral dental radiographs, such as panoramic radiographs or cone-beam C.T., as appropriate alternatives of intraoral radiography	ADA
		-If aerosol-generating procedures are inevitable for emergency care, use 4-handed dentistry.	CDC, ADA
		-Avoid the use of aerosol-generating procedures, handpieces/ultrasonic instruments, 3-in-1 syringes, and the air-water syringe whenever possible.	CDC, ADA
		-Dental professionals should use resorbable sutures to eliminate the need for a follow-up appointment.	ADA
		-Treatment should be completed in one visit wherever possible.	NHS
		-Environmental cleaning and disinfection procedures should be followed promptly after the completion of clinical care.	CDC
After dental treatment		-Clean PPE with soap and water, or if visibly soiled, clean and disinfect reusable facial protective equipment.	ADA
		-Manage laundry and medical waste in accordance with routine procedures.	CDC

According to the recommended guidelines during COVID-19, the protective measures that should be undertaken in a dental setting can be categorized into three phases: 1) prior to dental treatment, 2) during the dental procedure, and 3) after dental treatment.

### Pre-dental treatment

#### Before entering a dental office

Patient triage, identification of possible suspects, delay of non-urgent dental care, management of dental appointments, and active screening of dental staff are among the protective protocols that should be considered prior to the patient entering the dental office.

#### At the dental office

Active screening of patients, management of social distancing in the dental office, offering sanitation measures to the patients, use of facemasks by everyone entering the dental office, patient education, use of PPE by the dental team, and management of dental operatory room are among the procedures required to be carried out in dental offices.

### During dental treatment

Maintaining hand hygiene, offering a preoperative antimicrobial mouth rinse to patients, using rubber dams, high-volume saliva ejectors, and extraoral dental radiographs, using 4-handed dentistry, avoiding aerosol-generating procedures, one-visit treatment, and environmental cleaning and disinfection procedures should be implemented during dental procedures.

### Post dental treatment

Cleaning and disinfecting reusable facial protective equipment, as well as management of laundry and medical waste following routine procedures should be considered after dental treatment.

A large number of articles were identified considering the short duration of the search period. Due to the rapid development of the COVID-19 pandemic and the short publication timeframe, some published articles have subsequently been retracted or rejected and replaced with newer information. The publication of unreliable papers has several negative consequences that can increase the chance of incorrect treatment for patients [30]. In this study, an attempt was made to use more valid and practical articles and protocols.

## Possible risk of transmission of COVID-19 in dentistry

While it may be difficult to identify the particular mechanism of infection for individual patients, we are aware of the common routes of transmission. Droplet transmission and transmission through fomites (objects or materials which are likely to carry infection) are the main modes of transmission by the respiratory system in intrapersonal contact and especially during sneezing, dry coughing, or even talking [31]. Eye exposure has also been reported as a route of transmission for the virus, with infectivity even higher than that of SARS [32]. We also know that COVID-19 is present in saliva, but transmission through this route has not been conclusively confirmed [33]. Considering the main path of transmission of the COVID-19 disease, dental procedures that lead to the spray of saliva particles into the air (which means almost all dental procedures) could heighten the possibility of contamination [22]. Much effort has been made in the literature to define droplets and aerosols and to distinguish between their ability to carry the COVID-19 virus. Knowing which dental procedures produce aerosols that could carry the virus is important to help define the level of risk that these procedures create. This then helps to define what Personal protective equipment (PPE) is appropriate. As a result, both kinds of particles, or better to say, anything that comes out of the patient should be considered hazardous [34–36]. Given the fact that the majority of dental instruments are made from metal and polymers, the COVID-19 could adhere and persist on these surfaces for several days. Consequently, they could present a risk of virus transmission if they are not adequately decontaminated [31, 37]. Fundamentally, COVID-19 in dentistry may be transmitted through air, droplets, and contact [20, 38]. Not only could the professionals act as transmitters, but also, they could become infected during human-to-human transmission, through non-invasive salivary secretions like a patient’s cough or sneeze, or treatment procedures, such as using a high-speed handpiece or ultrasonic instruments which release aerosols which may contain saliva or blood bacteria and viruses into the environment. Therefore, using appropriate protective wearing is critical, given the fact that the spreading of saliva and dental fluids has the potential of virus transmission because of the close distance between patients and professionals [19, 39].

## Special precautions in dental procedure

PPE and hand hygiene should be given very serious attention in a dental clinic at all times, even when no patient is present [19, 40]. Regular hand hygiene could be regarded as a critical element in any controlling protocol to reduce the outbreak of infection [40]. Due to the fact that dentists have close contact with the patients and their hands are exposed to the mouth fluids and aerosols, using an antiseptic solution before treatment of each patient is of the utmost importance. Although broad types of antiseptic solutions are available, the ethanolic solutions (above 70% concentration) are suitable for this process because of the non-toxic entity, while ethanolic solutions are useful in hand hygiene; using soap and water is also effective [38]. Using masks with pores of less than 50 μm is necessary for dental professionals [39, 41, 42]. On the other hand, these particles could be transmitted through the eyes; therefore, using appropriate goggles or face shields could decrease the risk of the infections [23].

As the aerosols spread from the mouth, suggesting to patients, they use an antiseptic oxidative mouth rinse would be protective prior to and after treatment. Currently, ADA and CDC only recommend peroxide to eradicate the virus. Moreover, public health authorities have advised 0.2% chlorhexidine mouth-wash (CHX), 1% povidone-iodine (P.I.), 1.5% hydrogen peroxide (H_2_O_2_), or 0.05% hypochlorous acid (HOCl). CHX is weak in terms of virucidal, and the other three (P.I., H2O2, HOCl) all have excellent virucidal properties but are weak in substantivity, because saliva flow can potentially replace the virus. Clinically, the most acceptable in terms of virucidal and taste is 1.5% hydrogen peroxide [38, 43, 44].

## Discussion

The results of a study on 2537 patients showed that the nCoV-19 pandemic led to a decrease in the emergency dental treatment in Beijing, China, this is because the patients were reluctant to have dental treatment because of the potential risk of infection by going outside [45]. Oral hygiene and preventive practices have always been very important, but now, in the current condition, they are more critical than ever. Higher levels of oral hygiene could decrease the need for a person to attend a dental clinic for urgent matters; and at the same time, could significantly help the person to remove the virus from the body in the early contamination phase in day to day life [46] and also to reduce the bacterial load in the mouth and the risk of bacterial superinfection especially in patients who are prone to altered biofilms due to diabetes, high blood pressure or cardiovascular disease [47].

The COVID-19 pandemic has led to the closure of dental offices around the world. Some countries are currently re-opening or planning to re-open dental services. There are many protocols that need to be considered and integrated into a comprehensive and concise protocol (Tables 1 & 2). Smart appointment systems and generally avoiding crowding in dental clinics are vital [48]. Adequate time should be given between appointments so that appropriate decontamination procedures can be carried out [48].

## Specific therapeutic considerations in dentistry

Considering the time needed to develop a vaccine and the high possibility of SaRS-CoV-2 outbreak being around for a long time to come, throughout the world, there is an urge to define more distinct guidelines in order to reduce the risk of dental emergencies and at the same time offer urgent care to those patients in need. Suggestions have been made to preserve a high level of oral hygiene in patients in order to diminish the risk of any emergencies; including washing teeth at least twice a day, daily flossing, and using a 1% povidone-iodine mouth-wash 3 to 4 times a day [49].

### Endodontics and restorative dentistry

If emergency treatment is necessary, the ADA COVID-19 Dental Emergency document [42] recommends that chemomechanical caries removal and hand instrumentation should be prioritized over rotary systems. In the case of symptomatic irreversible pulpitis, reducing pain with a pulpotomy and pulpectomy or vital pulp therapy is recommended over conventional root canal therapies whenever possible [19, 50, 51].

### Periodontics

For periodontal treatments, priority should be given to manual scaling and polishing instead of ultrasonic techniques.

### Oral and maxillofacial surgery

In the case of tooth extraction, the use of high-volume saliva ejectors is crucial, preferably when the patient is in a supine position. If a suture is required, using absorbable material is advocated [37]. For the patients suffering extreme toothache and extensive caries, extraction of the pathogenic teeth could be considered instead of a restorative treatment as this could reduce the time of treatment and subsequently decrease the risk of infection [50, 51].

In the case of third molar abscesses or pericoronitis, antibiotic therapy (Azithromycin 500 mg P.O. per day for 3 to 5 days), a mouth-wash 3 times a day, as well as applying a chlorhexidine gel twice a day has been recommended over the infected area [49].

### Prosthodontics

For prosthodontic treatments, enhanced disinfection techniques of prosthetic materials and impressions are highly emphasized to minimize the risk of cross-contamination to prosthodontic laboratories. To avoid gag stimulation, salivary suction is recommended.

In the case of a cemented crown becoming loose, using a temporary cement that can be easily found in a drug store has been recommended to relieve the discomfort and, at the same time, inhibit the closure of the interocclusal and mesiodistal space.

Application of an over-the-counter soft removable liner in the case of a removable prosthesis causing discomfort can temporarily let the patient preserve function and esthetics.

### Oral and maxillofacial radiology

For diagnosis purposes, using extraoral radiographic such as Dental Panoramic Radiographs (DPRs) or Cone-Beam Computed Tomography (CBCT) are endorsed over the intraoral radiographs [19, 50].

As reducing face-to-face appointments is necessary to reduce the risk of infection, the Teledentistry provides an opportunity for many patients to access uninterrupted clinical and supportive care and the chance to triage increasingly critical conditions needing face-to-face clinic visits. Furthermore, Teledentistry allows for the continuing clinical training of dental practitioners [52–54]. The COVID-19 pandemic may cause a permanent transformation in dentistry with the advancement of Teledentistry [29]. In fact, the visual nature of dental procedures makes telemedicine practical in the dentistry field [55].

## Conclusion

This review focused on the methods, protocols, and recent reports regarding the nCoV-19 infection and the transmission process, which could occur during routine dental treatment and surgeries. While the currently available evidence has not demonstrated a clear and direct relationship between dental treatment or surgery and the possibility of the transmission of COVID-19, there is clearly the potential for transmission. This could result due to contaminated dental fluids, saliva, or aerosol spread during close human-to-human contact during dental treatment or by contact with contaminated instruments or surfaces. Therefore, following the previous literature, following the protective protocols in the COVID-19 crisis is of utmost importance in a dental setting. Although many articles have been published in journals during the Coronavirus 2019 crisis without appropriate methods and accurate judgment, careful and comprehensive research is recommended.

## Data Availability

The datasets used and analyzed during the current study are available from the corresponding author on reasonable request.

## Abbreviations

MESH: Medical Subject Headings
PPE: Personal Protective Equipment
WHO: The World Health Organization
CDC: The Centers for Disease Control and Prevention
NHS: The National Health Service
ADA: The American Dental Association
AHDA: American Dental Hygienists’ Association
Tx: Stands for treatment
Hx: Stands for history
ABHR: Alcohol-based hand rub
CHX: Chlorhexidine mouth-wash
P.I.: 1% povidone-iodine
H_2_O_2_: 1.5% hydrogen peroxide
HOCl: 0.05% hypochlorous acid
CBCT: Cone-beam computed tomography
DPRs: Dental panoramic radiographs

## References

[1] Letko M, Marzi A, Munster V (2020). Functional assessment of cell entry and receptor usage for SARS-CoV-2 and other lineage B betacoronaviruses. Nat Microbiol.

[2] Sohrabi C, Alsafi Z, O’Neill N, Khan M, Kerwan A, Al-Jabir A (2020). World Health Organization declares global emergency: a review of the 2019 novel coronavirus (COVID-19). Int J Surg.

[3] World Health Organization (2020). Novel coronavirus (2019-nCoV) situation reports.

[4] Passarelli PC, Lopez MA, Bonaviri GNM, Garcia-Godoy F, D'Addona A (2020). Taste and smell as chemosensory dysfunctions in COVID-19 infection. Am J Dent.

[5] Ali S, Zeb U, Khan M, Muhammad A (2020). Transmission routes and infection control of novel Coronavirus-2019 in dental clinics–a review. J Islamabad Med Dent Coll.

[6] Lai C-C, Shih T-P, Ko W-C, Tang H-J, Hsueh P-R (2020). Severe acute respiratory syndrome coronavirus 2 (SARS-CoV-2) and corona virus disease-2019 (COVID-19): the epidemic and the challenges. Int J Antimicrob Agents.

[7] Napimoga MH, FREITAS ARRdd. Dentistry vs severe acute respiratory syndrome coronavirus 2: how to face this enemy. RGO-Revista Gaúcha de Odontol 2020;68:1. 10.1590/1981-863720200001120200034.

[8] Wadia R (2020). Transmission routes of COVID-19 in the dental practice. Br Dent J.

[9] Gamio L (2020). The workers who face the greatest coronavirus risk. New York times.

[10] Coulthard P (2020). The oral surgery response to coronavirus disease (COVID-19). Keep calm and carry on?. Oral Surg.

[11] Krithikadatta J, Nawal RR, Amalavathy K, McLean W, Gopikrishna V (2020). Endodontic and dental practice during COVID-19 pandemic: position statement from the Indian endodontic society, Indian dental association, and International Federation of Endodontic Associations. Endodontology..

[12] Holohan T, Ghebreyesus TA (2020). COVID-19 and dental practice in Ireland.

[13] Farooq I, Ali S. COVID-19 outbreak and its monetary implications for dental practices, hospitals and healthcare workers. Postgrad Med J. 2020. 10.1136/postgradmedj-2020-137781. 10.1136/postgradmedj-2020-137781 PMC10016845 32245754

[14] Ather A, Patel B, Ruparel NB, Diogenes A, Hargreaves KM (2020). Coronavirus disease 19 (COVID-19): implications for clinical dental care. J Endod.

[15] Alharbi A, Alharbi S, Alqaidi S (2020). Guidelines for dental care provision during the COVID-19 pandemic. Saudi Dent J.

[16] Izzetti R, Nisi M, Gabriele M, Graziani F (2020). COVID-19 transmission in dental practice: brief review of preventive measures in Italy. J Dent Res.

[17] Lee YH, Auh QS. Strategies for prevention of coronavirus disease 2019 in the dental field. Oral Dis. 2020. 10.1111/odi.13361. 10.1111/odi.13361 PMC7264497 32306496

[18] Mallineni SK, Innes NP, Raggio DP, Araujo MP, Robertson MD, Jayaraman J (2020). Coronavirus disease (COVID-19): characteristics in children and considerations for dentists providing their care. Int J Paediatr Dent.

[19] Meng L, Hua F, Bian Z (2020). Coronavirus disease 2019 (COVID-19): emerging and future challenges for dental and oral medicine. J Dent Res.

[20] Peng X, Xu X, Li Y, Cheng L, Zhou X, Ren B (2020). Transmission routes of 2019-nCoV and controls in dental practice. Int J Oral Sci.

[21] Prati C, Pelliccioni G, Sambri V, Chersoni S, Gandolfi M (2020). COVID-19: its impact on dental schools in Italy, clinical problems in endodontic therapy and general considerations. Int Endod J.

[22] Spagnuolo G, De Vito D, Rengo S, Tatullo M (2020). COVID-19 outbreak: an overview on dentistry. Multidiscipl Digit Publishing Inst.

[23] World Health Organization. Rational use of personal protective equipment for coronavirus disease ( COVID-19) and considerations during severe shortages: interim guidance, April 6 2020. 2020; Available from: https://apps.who.int/iris/bitstream/handle/10665/331695/WHO-2019-nCov-IPC_PPE_use-2020.3-ara.pdf. [Accessed 6 April 2020].

[24] Association A.D. (2020). ADA guideline.

[25] Association ADH (2020). ADHA guideline.

[26] Health CO (2020). Recommendations for the re-opening of dental services: a rapid review of international sources.

[27] Prevention CfDCa (2020). CDC guidance for providing dental care during COVID-19.

[28] Centers for Disease Control and Prevention (2020). Interim infection prevention and control guidance for dental settings during the COVID-19 response.

[29] World Health Organization (2020). Coronavirus disease (COVID-19) technical guidance: infection prevention and control / WASH, WHO.

[30] Rapani A, Lombardi T, Berton F, Del Lupo V, Di Lenarda R, Stacchi C (2020). Retracted publications and their citation in dental literature: a systematic review. Clin Exp Dent Res.

[31] Lauer SA, Grantz KH, Bi Q, Jones FK, Zheng Q, Meredith HR (2020). The incubation period of coronavirus disease 2019 (COVID-19) from publicly reported confirmed cases: estimation and application. Ann Intern Med.

[32] Passarelli PC, Rella E, Manicone PF, Garcia-Godoy F, D’Addona A (2020). The impact of the COVID-19 infection in dentistry. Exp Biol Med.

[33] Tsang OT-Y, Yip CC-Y, Chan K-H, Wu T-C, Chan JM-C, To KK-W (2020). Consistent detection of 2019 novel coronavirus in saliva. Clin Infect Dis.

[34] Wang S, Liu K, Yao X, Jiang L (2015). Bioinspired surfaces with superwettability: new insight on theory, design, and applications. Chem Rev.

[35] Khalil-Abad MS, Yazdanshenas ME (2010). Superhydrophobic antibacterial cotton textiles. J Colloid Interface Sci.

[36] Chenab KK, Sohrabi B, Rahmanzadeh A (2019). Superhydrophobicity: advanced biological and biomedical applications. Biomater Sci.

[37] Negahdaripour M (2020). The battle against COVID-19: where do we stand now?. Iranian J Med Sci.

[38] Kampf G, Todt D, Pfaender S, Steinmann E (2020). Persistence of coronaviruses on inanimate surfaces and their inactivation with biocidal agents. J Hosp Infect.

[39] Sabino-Silva R, Jardim ACG, Siqueira W.L. Coronavirus COVID-19 impacts to dentistry and potential salivary diagnosis. Clin Oral Investig 2020;24(4):1619–1621. 10.1007/s00784-020-03248-x. 10.1007/s00784-020-03248-x PMC7088419 32078048

[40] Mathur N, Tyagi S, Dwivedi V, Narang A, Tyagi P, Nath KS (2020). Dental considerations amidst COVID-19 scare. Int J Med Biomed Stud.

[41] Xie X, Li Y, Sun H, Liu L (2009). Exhaled droplets due to talking and coughing. J Royal Soc Interface.

[42] Dutil S, Mériaux A, de Latrémoille M-C, Lazure L, Barbeau J, Duchaine C (2008). Measurement of airborne bacteria and endotoxin generated during dental cleaning. J Occup Environ Hyg.

[43] Khader Y, Al Nsour M, Al-Batayneh OB, Saadeh R, Bashier H, Alfaqih M (2020). Dentists’ awareness, perception, and attitude regarding COVID-19 and infection control: cross-sectional study among Jordanian dentists. JMIR Public Health Surveill.

[44] M-S H (2020). Mouthwash; can it reduce levels of Covid-19 in the mouth? : Nationalelfservice.

[45] Guo H, Zhou Y, Liu X, Tan J. The impact of the COVID-19 epidemic on the utilization of emergency dental services. J Dent Sci. 2020. 10.1016/j.jds.2020.02.002. 10.1016/j.jds.2020.02.002 PMC7156222 32296495

[46] Lucaciu O, Tarczali D, Petrescu N. Oral healthcare during the COVID-19 pandemic. J Dent Sci. 2020. 10.1016/j.jds.2020.04.012. 10.1016/j.jds.2020.04.012 PMC7252092 32837682

[47] Sampson V (2020). Oral hygiene risk factor. Br Dent J.

[48] Ren Y, Rasubala L, Malmstrom H, Eliav E (2020). Dental care and oral health under the clouds of COVID-19. JDR Clin Transl Res.

[49] Santacroce L, Passarelli PC, Passarelli G, Charitos IA, Rella E, D’Addona A (2020). COVID-19 and Oral diseases: how can we manage hospitalized and quarantined patients while reducing risks?.

[50] Shamszadeh S, Parhizkar A, Mardani M, Asgary S. Dental considerations after the outbreak of 2019 novel coronavirus disease: a review of literature. Arch Clin Infect Dis. 2020, 15:2. 10.5812/archcid.103257.

[51] Dave M, Seoudi N, Coulthard P. Urgent dental care for patients during the COVID-19 pandemic. Lancet. 2020;395(10232):1257. 10.1016/S0140-6736(20)30806-0. 10.1016/S0140-6736(20)30806-0 PMC7270877 32251619

[52] Maret D, Peters OA, Vaysse F, Vigarios E (2020). Integration of telemedicine into the public health response to COVID-19 must include dentists. Int Endod J.

[53] Health UDo, Services H. Notification of enforcement discretion for telehealth remote communications during the COVID-19 nationwide public health emergency. Retrieved on March. 2020;27:2020. Available from: https://www.hhs.gov/hipaa/for-professionals/special-topics/emergency-preparedness/notification-enforcement-discretion-telehealth/index.html. [Accessed 30 Mar 2020].

[54] Villa A, Sankar V, Shiboski C. Tele (oral) medicine: a new approach during the COVID-19 crisis. Oral Dis. 2020. 10.1111/odi.13364. 10.1111/odi.13364 PMC7264524 32307831

[55] Estai M, Kanagasingam Y, Xiao D, Vignarajan J, Huang B, Kruger E (2016). A proof-of-concept evaluation of a cloud-based store-and-forward telemedicine app for screening for oral diseases. J Telemed Telecare.

